# Effects of light-emitting diode irradiation on the osteogenesis of human umbilical cord mesenchymal stem cells in vitro

**DOI:** 10.1038/srep37370

**Published:** 2016-11-22

**Authors:** Dazhi Yang, Weihong Yi, Ertian Wang, Min Wang

**Affiliations:** 1 Department of Orthopaedics, Nanshan Hospital, Guangdong MedicalCollege, Shenzhen Guangdong, 518052, China

## Abstract

The aim of this study was to examine the effects of light-emitting diode (LED) photobiomodulation therapy on the proliferation and differentiation of human umbilical cord mesenchymal stem cells (hUMSCs) cultured in osteogenic differentiation medium. HUMSCs were irradiated with an LED light at 620 nm and 2 J/cm^2^ and monitored for cell proliferation and osteogenic differentiation activity. The experiment involved four groups of cells: the control group; the osteogenic group (osteo group); the LED group; the osteogenic + LED group (LED + osteo group). HUMSC proliferation was detected by performing a3-(4,5-dimethylthiazol-2yl)-2,5 diphenyltetrazolium bromide(MTT) assay. Osteogenic activity was evaluated by performing alkaline phosphatase (ALP) and Von Kossa staining, and osteopontin (OPN) gene mRNA expression was evaluated byreverse transcription polymerase chain reaction (RT-PCR). The hUMSCs in the LED + osteo group exhibited a significantly higher proliferation rate than the other subgroups. Additionally, there were greater numbers of ALP-positive cells and Von Kossa nodules in the LED + osteo group. OPN mRNA expression in the LED + osteo group was higher than other subgroups. In conclusion, low levels of LED light at a wavelength of 620 nm enhance the proliferation and osteogenic differentiation of hUMSCs during a long culture period.

Photobiomodulation therapyis broadly applied in different medical fields, including oral surgery and wound healing^1^,^2^,^3^. According to a recent report, photobiomodulation therapy with a helium-neon (He-Ne) laser increases the number of chondrocytes *in vitro* and induces cartilage neoformation^4^. Increased proliferation after low-power laser irradiation has been observed for many cell types *in vitro*, including fibroblasts, human osteoblasts, calvaria osteoblast-like cells, and mesenchymal stem cells (MSCs)^5^,^6^.

In addition to He-Ne lasers, light-emitting diodes (LEDs) activate cells and tissues. Several studies have investigated the effects of LEDs of various wavelengths on the osteogenic differentiation of amniotic fluid-derived stem cells (ASFCs). The osteogenic differentiation of ASFCs is facilitated by green (525 nm) and blue (470 nm) light irradiation^7^. Additionally, red LED light promotes the osteogenic differentiation of bone marrow mesenchymal stem cells (BMSCs) in osteogenic differentiation medium (ODM)^8^ and increases the proliferation of osteoblast-like MC3TC-E1 cells^9^. Low-levellight regulates cellular processes such as mitochondrial function and mitosis, likely by mediating light-induced reactive oxygen species (ROS)^10^. Light is also absorbed by mitochondrial respiratory chain components, resulting in increased levels of adenosine triphosphate and cyclic AMP, which promote cellular proliferation and cytoprotection^11^.

However, few reports have examined the effects of red LED light on osteogenic differentiation in human umbilical cord mesenchymal stem cells (hUMSCs) and no study has investigated long-term changes induced by LED irradiation. hUMSCs differ from BMSCs with respect to mitochondrial function and energy metabolism^12^. Therefore, the purpose of this study was to investigate the effects of red LED light with a wavelength of 620 nm on the proliferation and differentiation of hUMSCs cultured in ODM.

## Results

### Cell proliferation as determined by 3-(4,5-dimethylthiazol-2yl)-2,5 diphenyltetrazolium bromide (MTT) assays

**Figure 1:**
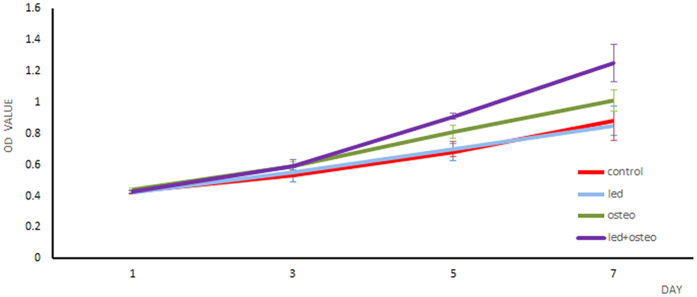
hUMSC proliferation in the control group, LED group, osteo group and LED + osteo group. The cell proliferation rates of all groups over time are shown. The highest proliferation was observed on day 7 for all groups. The osteo and LED + osteo groups demonstrated significant increases compared with the other two groups on days 5 and 7. The LED + osteo group had the highest OD values among all groups on days 5 and 7.

There were significant increases in the cell proliferation rates of all groups over time. The highest proliferation was observed on day 7 for all groups, as shown in Fig. 1. The optical density (OD) scores of the control group were 0.68 ± 0.05 on day 5 and 0.88 ± 0.093 on day 7, and the LED group scores were 0.70 ± 0.053 on day 5 and 0.85 ± 0.094 on day 7; these scores did not differ significantly between groups(p > 0.05). By contrast, the osteo and LED + osteo groups demonstrated significant increases compared with the other two groups on days 5 and 7 (p < 0.05). Additionally, the OD values of the LED + osteo group reached 0.91 ± 0.021 on day 5 and 1.25 ± 0.12 on day 7, which were significantly higher thanall the other groups (p < 0.05).

### Alkaline phosphatase (ALP) staining

**Figure 2:**
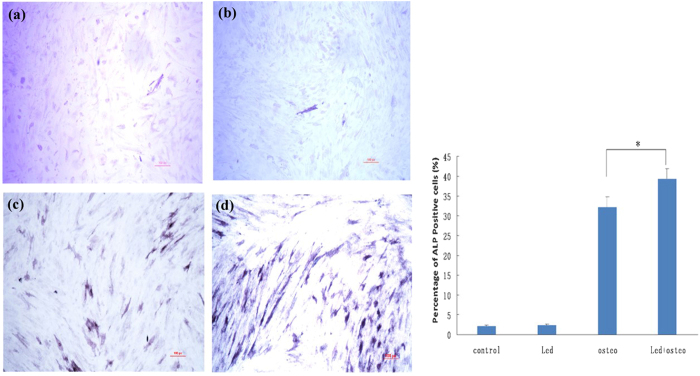
ALP staining of cultured hUMSCs in the control group (**a**), osteo group (**b**), LED group (**c**), and LED + osteo group (**d**). Few positive cells in the control group and LED group were detected, whereas many positive cells were observed in the osteo group (**c**) and the LED + osteo group (**d**). The scale bar indicates 100 μm.

ALP staining was performed to examine the osteogenic differentiation status of hUMSCs. After 14 days of induced differentiation, there were few positive purple cells in the control group and LED group (Fig. 2). Some positive cells containing intracellular purple granular dye were observed in the osteo group (Fig. 2b), whereas we observed greater numbers of ALP-positive cells (Fig. 2c) in the LED + osteo group than in the osteo group. Quantitative analysis indicated the percentages of ALP-positive cells in the control group, LED group, osteo group and LED + osteo group to be 2.19 ± 0.29%, 2.32 ± 0.22%, 32.2 ± 2.52%, and 39.35 ± 2.55%. The percentage of ALP-positive cells in the LED + osteo group was significantly higher than that in the osteo group (p < 0.05) (Fig. 2).

### Von Kossa staining

**Figure 3:**
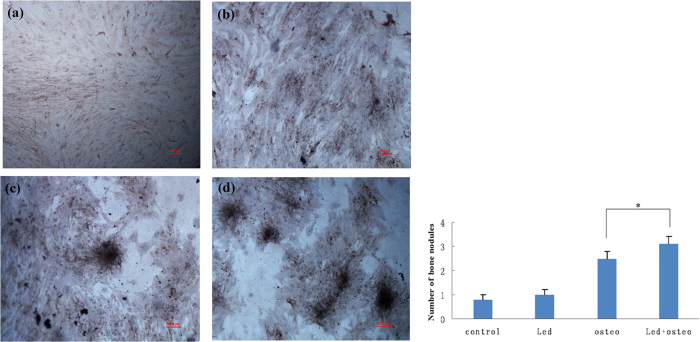
Von Kossa staining of hUMSCs in the control group (**a**), osteo group (**b**), LED group (**c**), and LED + osteo group (**d**). Few bone nodules were detected in the control group (**a**) and LED group (**b**), and low numbers of bone nodules were observed in the osteo group (**c**), whereas greater numbers of mineralized bone nodules were found in the LED + osteo group (**d**). The number of nodules in the LED + osteo group was significantly higher than that of the osteo group (p < 0.05). The scale bar indicates 100 μm.

After 4 weeks of treatment, Von Kossa staining revealed few bone nodules in the control group and LED group (Fig. 3a,b). Low numbers of bone nodules were also observed in the osteo group (Fig. 3c), but greater numbers of mineralized bone nodules were found in the LED + osteo group (Fig. 3d). Quantification indicated 0.8 ± 0.2, 1.0 ± 0.2, 2.5 ± 0.3, and 3.1 ± 0.3 mineralized calcium nodules per high-magnification field in the control, LED, osteo and LED + osteo groups, respectively. The number of nodules in the LED + osteo group was significantly higher than that of the osteo group (p < 0.05).

### Osteopontin (OPN) mRNA expression

**Figure 4:**
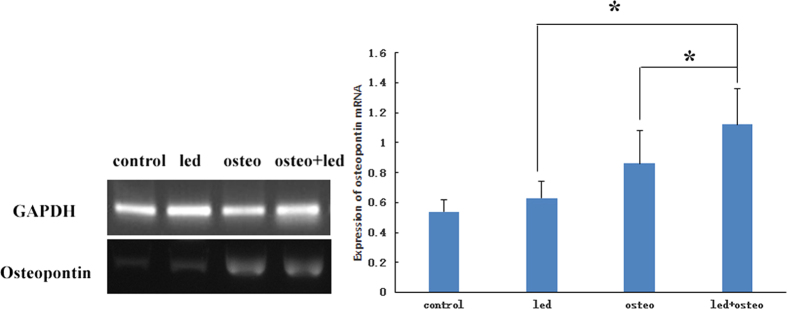
OPN mRNA expression in the hUMSCs of the control group, osteo group, LED group, and LED + osteo group. Both the osteo group and LED + osteo group demonstrated increased OPN mRNA expression. OPN mRNA expression in the LED + osteo group was significantly higher than those of the control group, LED group and osteo group (p < 0.05).

Reverse transcription polymerase chain reaction(RT-PCR) was performed to analyze changes in OPN gene expression in all four groups after 2 weeks of culture. We observed increases in OPN mRNA expression of 0.86 ± 0.22% and 1.12 + 0.24% in the osteo group and the LED + osteo group, respectively, which were significantly higher than the control group (0.54 ± 0.08) and the LED group (0.63 ± 0.11) (Fig. 4). Furthermore, the LED + osteo group demonstrated higher expression than all the other groups (p < 0.05).

## Discussion

In this study, we evaluated the proliferation and osteogenic differentiation of hUMSCs that were cultured in osteogenic medium after irradiation with an LED light at a wavelength of 620 nm. As hypothesized, culture in osteogenic medium with an LED light at a wavelength of 620 nm enhanced the osteogenic differentiation of hUMSCs.

Low-power laser irradiation promotes the proliferation and differentiation of osteogenic cell lines, such as MC3T3-E1 cells^13^. Although lasers emit coherent light and LEDs emit non-coherent light, both induce similar photobiological effects when used with the same irradiation parameters^5^. According to a previous report, LED energy density extends over a wide range of 0.2–10 J/cm^2^ and is not defined. LED irradiation at 630 nm promotes OPN mRNA expression in MC3T3-E1 cells^9^, but the molecular mechanisms underlying photobiomodulation therapy have remained unclear. All hMSC types exhibit different energy metabolism levels and behavior. hUMSCs possess higher mitochondrial superoxide and manganese superoxide dismutase (SOD) levels than BMSCs^14^.Low-levellight regulates cellular processes such as mitochondrial function and mitosis, which are likely mediated by light-induced reactive oxygen species (ROS). Specifically, low-level laser irradiation induces the production of ROS, which are important secondary messengers that regulate the activities of various protein kinases^15^. The Src kinases, which are non-receptor tyrosine kinases, are well-known targets of ROS and are activated by oxidative events. Src activation via light irradiation occurs in a dose-dependent manner, and in the presence of vitamin C or SOD, Src activation, which is mediated by ROS, is significantly abolished^16^; thus, light irradiation-induced changes in cell viability may occur via the ROS/Src pathway. Light irradiation causes increases in membrane potential, ATP, NADH and cAMP; cAMP elevation in turn stimulates both DNA and RNA synthesis^17^. The ATP/cAMP pathway regulates light irradiation-induced cell proliferation. Furthermore, light irradiation regulates cellular homeostasis parameters, such as the redox state of a cell and the expression of redox-sensitive factors such as NF-κB, which lead to proliferation^18^. Several genes related to antioxidation and mitochondrial energy metabolism are up-regulated or down-regulated in response to light irradiation^19^. The MAPK/ERK pathway is likely responsible for the activation of signaling pathways resulting in the differentiation of ASFCs into osteogenic cells upon light irradiation^20^.

In this study, the LED group did not demonstrate a significant increase in hUMSC proliferation and osteogenic differentiation, but irradiation with an LED light at a wavelength of 620 nm in combination with osteogenic medium did dramatically promote hUMSC proliferation and osteogenic differentiation. During the process of osteogenic differentiation, LED light at 620 nm in combination with osteogenic medium upregulated ALP expression and enhanced calcium deposition, which was accompanied by bone nodule formation. After 14 days of induced differentiation, the percentage of ALP-positive cells in the LED + osteo group was significantly higher than that in the LED and osteo groups (p < 0.05). Additionally, the number of nodules in the LED + osteo group was significantly higher than that of the LED and osteo groups (p < 0.05) after 28 days in culture. OPN is an important marker of osteogenic differentiation^9^. RT-PCR indicated changes in OPN gene expression for all four groups at 2 weeks, and the LED + osteo group demonstrated increased expression compared with the other groups (p < 0.05). LED light at 620 nm alone did not directly promote hUMSC proliferation and osteogenic differentiation, but hUMSC proliferation and osteogenic differentiation were enhanced following culture in osteogenic medium in combination with LED light. These results differed from those of previous studies that investigated the effects of LED light on BMSCs. These differences are potentially attributable to differences between hUMSCs and BMSCs. Different types of MSCs exhibit different energy metabolism levels. hUMSCs have higher mitochondrial superoxide and manganese SOD levels than BMSCs^15^. However, LED light at 620 nm in combination with osteogenic medium clearly played a role in promoting proliferation and osteogenic differentiation.

In conclusion, LED light irradiation at a wavelength of 620 nm did not directly promote hUMSC proliferation and osteogenic differentiation. However, LED light irradiation at a wavelength of 620 nm enhanced the proliferation and osteogenic differentiation of hUMSCs when they were cultured in osteogenic medium. Elucidation of the mechanisms underlying LED irradiation will potentially suggest applications for LED light irradiation in bone tissue regeneration and repair.

## Materials and Methods

### Ethics statement

All experiments were performed in accordance with the guidelines provided by the Medical Central Laboratory of Guangdong MedicalCollege and approved by the Human Ethics Committee of Guangdong MedicalCollege. Written informed consent was obtained from all patients at the time of admission to authorize the use of blood, tissue and other samplesfor scientific purposes.

### Cell culture

hUMSCs were isolated from umbilical cords with patient consent according to the methods described by Fan Yang^21^ and generously provided by the Shenzhen Beike Stem Cell Engineering Institute. The cells were cultured in Dulbecco’s modified Eagle’s medium (DMEM, Gibco, California) supplemented with 10% fetal bovine serum (FBS, Hyclone) and 1% (v/v) penicillin–streptomycin solution (50 U/ml penicillin and 50 mg/ml streptomycin; Sigma Chemical, St. Louis, Missouri) at 37 °C in a humidified atmosphere containing 95% air/5% CO_2_. The culture medium was refreshed every 3 days before the cells reached confluence. Phase contrast images were obtained using a microscope (IX71, Olympus, Japan). The osteogenic medium consisted of DMEM supplemented with 10^−5^ mM dexamethasone, 0.2 mM ascorbic acid-phosphate, and 10 mM glycerol 2-phosphate (Sigma). The medium was changed three times per week for each group.

The experiment involved four groups of cells: the control group was cultured in Dulbecco’s modified Eagle’s medium (DMEM) + 10% fetal bovine serum (FBS); the osteogenic group (osteo group) was cultured in osteogenic medium + 10% FBS; the LED group was cultured in DMEM + 10% FBS + LED light (2 J/cm2); the osteogenic + LED group (LED + osteo group) was cultured in osteogenic medium + 10% FBS + LED light (2 J/cm2).

### LED irradiation procedure

**Figure 5:**
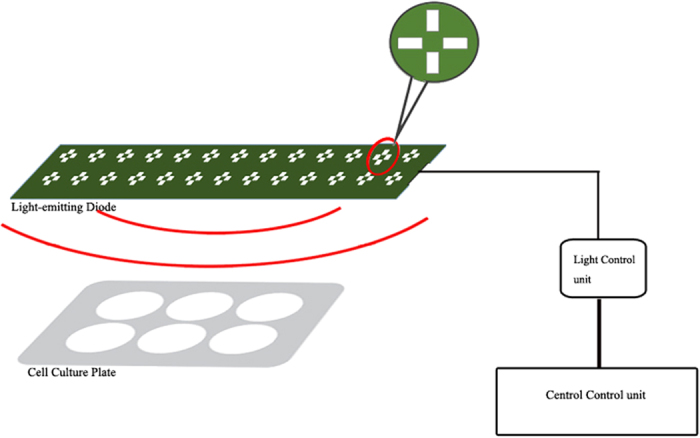
Image of the experimental device arrangement for 620-nm LED irradiation *in vitro*. The device consisted of three parts: an integrated circuit plate upon which a 620-nm LED was fixed, a light control unit, and a central control unit. Cell culture plates were irradiated with a 620-nm LED laser at an energy density of 2 J/cm^2^ in a pulse-wave mode of operation at a 10-cm distance.

HUMSCs were plated in 96-well plates at a density of 2 × 10^4^ cells/cm^2^. After 24 hours of incubation to achieve adhesion, the cells were irradiated with an LED laser at a wavelength of 620 nm and an energy density of 2 J/cm^2^ and 50 Hz in a pulse-wave mode of operation at a distance of 10 cm. Light was delivered for 15 minutes every 8 hours. The620-nm LED bulb (Zhongshan Hoprled Optoelectronics Technology Company, China) was fixed on an integrated circuit plate, which was controlled by a light control unit and a central control unit (Fig. 5). The power density of the laser beam was measured with a laser power meter (Ophir, Israel).

### MTT assay

A total of 1 × 10^4^ cells per well were seeded in a 96-well plate and cultured. The cell culture medium was exchanged after 4 hours for different 4 types of medium: control group medium, osteo group medium, LED group medium and LED + osteo group medium. The LED group was cultured in DMEM with 10% FBS and irradiated with an LED laser. The LED + osteo group was cultured inosteogenic medium with 10% FBS and irradiated with an LED laser. For both the LED groupand the LED + osteo group, the LED laser had a wavelength of 620 nm and an energy density of 2 J/cm^2^ at 50 Hz. Light was delivered for 15 minutes every 8 hours. MTT assays were performed to measure viability for all experimental groups on days 1, 3, 5 and 7. The MTT (Sigma) was dissolved at a concentration of 5 mg/ml in sterile phosphate-buffered saline (PBS), filtered through a 0.22-pm filter to remove any formazan crystals, and stored at 4 °C in the dark. The MTT solution was added to the plates at a 1:10 ratio. Following incubation at 37 °C for 4 hours in a humidified atmosphere containing 5% CO_2_/95% air, supernatants were removed from the wells and replaced with 150 μl of dimethyl sulfoxide per well. The absorbance was measured at a wavelength of 490 nm using a microplate reader (Sunrise, TECAN, Switzerland). OD values were obtained to indicate the percent viability of the samples.

### ALP staining

A total of 2 × 10^4^ cells per well were seeded in 6-well plates and cultured for 14 days with 4 types of medium: control group medium, osteo group medium, LED group medium and LED + osteo group medium. For the LED groupand the LED + osteo group, LED laser irradiation was applied for 20 minutes every 8 hoursat a wavelength of 620 nm and an energy density of 2 J/cm^2^. The ALP staining procedure was carried out according to the manufacturer’s protocol for the BCIP/NBT ALP Kit (Beyotime, China). Briefly, the samples were incubated in a mixture of nitro-blue tetrazolium and 5-bromo-4-chloro-3-indoly1-phosphate for 1 hour after fixation in 4% neutral formalin for 30 minutes. There were five wells per group, and 3 pictures were randomly obtained for each culture well. ALP activity was assessed by counting the number of cells containing granular dye deposits that were blue in color. The percent of ALP-positive cells was calculated for each group and compared with the control group.

### OPN mRNA expression determined by RT-PCR

A total of 2 × 10^4^ cells per well were seeded in 6-well plates and cultured in 4 types of medium: control group medium, osteo group medium, LED group medium and LED + osteo group medium. For the LED groupand LED + osteo group, LED laser irradiation was applied for 20 minutes every 8 hoursat a wavelength of 620 nm and an energy density of 2 J/cm^2^. After 2 weeks of culture, total RNA was extracted from the cells of all 4 groups using TRIzol reagent. The procedure was carried out according to the manufacturer’s protocol for the PrimeScript® One Step RT-PCR Kit (Takara, Japan). Primer sequences and product sizes are shown in Table 1. Briefly, RNA was transcribed at 50 °C for 30 minutes, followed by denaturation at 94 °C for 30 seconds, annealing at 55°Cfor 30 seconds and extension at 72 °C for 30 seconds for a total of 35 cycles. The PCR products were fractionated in agarose gels. Gels were scanned under ultraviolet (UV) light and detected with a densitograph system (Dolphin Doc Plus, Taiwan). GAPDH was used as an internal standard to normalize RNA concentrations. Band densities were semi-quantified and normalized to GAPDHusing Image J software (version 1.36, National Institutes of Health).

### Von Kossa staining

A total of 2 × 10^4^ cells per well were seeded in 6-well plates and cultured for 28 days in 4 types of medium. For the LED groupand the LED + osteo group,LED laser irradiation was applied for 15 minutes every 6 hoursat a wavelength of 620 nm and an energy density of 2 J/cm^2^. All groups of cells were fixed in 4% buffered formalin for 30 minutes and rinsed 3 times with PBS. Then, 5% silver nitrate (AgNO_3_) was added. The plates were positioned under UV light for 1.5 hours in the dark. The cells were rinsed 3 times with tap water and treated with 5% sodium thiosulfate to remove background stain. After washing 3 times, the dishes were finally air-dried prior to microscopy. There were 3 wells per group, and 3 pictures were randomly obtained for each culture well. The number of bone nodules per high-magnification field was calculated for and compared among the 4 groups.

### Statistical analysis

Results were expressed as the mean and standard deviation (SD). The data showed a parametric distribution; thus, Student’s t-test was used to compare the results from the normal and LED-irradiated groups. All statistical tests were performed using Stata (version 13.0, www.stata.com). A p value ≤ 0.05 was considered statistically significant.

## Additional Information

**How to cite this article**: Yang, D. *et al*. Effects of light-emitting diode irradiation on the osteogenesis of human umbilical cord mesenchymal stem cells in vitro. *Sci. Rep.* **6**, 37370; doi: 10.1038/srep37370 (2016).

**Publisher’s note:** Springer Nature remains neutral with regard to jurisdictional claims in published maps and institutional affiliations.

## Figures and Tables

**Table 1:** Primers for RT-PCR analysis.

Gene	Forward primer 5′ to 3′	Product size(bp)
Osteopontin	AAGCTTCCATGGGAATTAGAGTGATTTGCTTTTGCCTC (Forward) GGATCCTTAATTGACCTCAGAAGATGCACTATCTAA (Reverse)	904
GAPDH	TCCCTCTTCCCTCCTCAAATTC TCAGCGTGTAAAGGCATCTG (Reverse)	465
